# A workforce survey of Australian chiropractic: the profile and practice features of a nationally representative sample of 2,005 chiropractors

**DOI:** 10.1186/s12906-016-1542-x

**Published:** 2017-01-05

**Authors:** Jon Adams, Romy Lauche, Wenbo Peng, Amie Steel, Craig Moore, Lyndon G. Amorin-Woods, David Sibbritt

**Affiliations:** 1 Australian Research Centre in Complementary and Integrative Medicine (ARCCIM), University of Technology Sydney, Level 8, Building 10, 235-253 Jones Street, Ultimo, NSW 2007 Australia; 2 Office of Research, Endeavour College of Natural Health, Brisbane, Australia; 3 School of Health Professions, Murdoch University, Murdoch, Australia

**Keywords:** Chiropractic, Chiropractor, Complementary and alternative medicine, Workforce, Practice-based research network

## Abstract

**Background:**

This paper reports the profile of the Australian chiropractic workforce and characteristics of chiropractic care from a large nationally-representative sample of practitioners.

**Methods:**

A 21-item questionnaire examining practitioner, practice and clinical management characteristics was distributed to all registered chiropractors (*n* = 4,684) in Australia in 2015 via both online and hard copy mail out.

**Results:**

The survey attracted a response rate of 43% (*n* = 2,005), and the sample is largely representative of the national chiropractic workforce on a number of key indicators. The average age of the chiropractors was 42.1 years, nearly two-thirds are male, and the vast majority hold a bachelor degree or higher qualification. Australian chiropractors are focused upon treating people across a wide age range who mainly present with musculoskeletal conditions. Australian chiropractors have referral relationships with a range of conventional, allied health and complementary medicine (CAM) providers.

**Conclusion:**

The chiropractic profession represents a substantial component of the contemporary Australian health care system with chiropractors managing an estimated 21.3 million patient visits per year. While the Australian chiropractic workforce is well educated, research engagement and research capacity remains sub-optimal and there is much room for further capacity building to help chiropractic reach full potential as a key integrated profession within an evidence-based health care system. Further rich, in-depth research is warranted to improve our understanding of the role of chiropractic within the Australian health care system.

## Background

Chiropractic constitutes a substantial component of health care seeking in Australia and worldwide [1, 2], with a 12-month utilisation prevalence of 16% in Australia [2]. In 2014, the chiropractic profession had a registered workforce of 4,684 practitioners in Australia [3] represented by two major organisations — the Chiropractors’ Association of Australia (CAA) and the Chiropractic and Osteopathic College of Australasia (COCA). Annual expenditure on chiropractic care (alone or combined with osteopathy) in Australia is estimated to be between AUD$750-988 million [2, 4, 5] with musculoskeletal complaints such as back and neck pain making up the bulk of consultations [2, 6–8]; and proportional expenditure is similar to that found in other countries [9–13]. While Medicare (the Australian publicly funded universal health care scheme) coverage of chiropractic services is limited to only those directed by a medical referral to assist chronic disease management [14], most private health insurers in Australia do provide partial reimbursement for a wider range of chiropractic services [15] in addition to limited third party payments for workers compensation and motor vehicle accidents.

As for the Australian workforce characteristics of the chiropractic profession, previous surveys show that the majority of chiropractors are male (64–67%) and aged between 25 and 54 years (80.3%); with 16% of practitioners aged 55 years or older [3, 16]. In addition, the majority (>90%) of chiropractors in Australia are university educated and hold a bachelor or postgraduate degree [1]. The vast majority of Australian chiropractors work full-time dedicating 33 working hours per week on average [16]. While previous Australian surveys have provided important insights into basic chiropractic workforce characteristics, they have often lacked rich, in-depth examination of core aspects of practice and clinical management characteristics [1, 16]; and/or have drawn upon small local samples raising doubts around the generalisability of their results [17].

Yet, in-depth, national Australian chiropractic workforce data is vital for helping understanding the existing workforce, supporting future workforce planning, and helping determine if chiropractic training, education and research adequately reflect the practice characteristics and techniques/methods used in grass-roots daily routine care. Moreover, such workforce data also helps appreciate the current and potential interface of chiropractors with other health providers and the role of chiropractors within the wider Australian health system.

In direct response to this significant research gap, this paper provides an in-depth examination of the profile of the Australian chiropractic workforce and characteristics of chiropractic care from a large nationally-representative sample of practitioners.

## Methods

This paper reports analyses from a questionnaire distributed as part of the recruitment for the Australian Chiropractic Research Network (ACORN) project — a national practice-based research network (PBRN) independently designed and conducted by senior researchers at the Australian Research Centre in Complementary and Integrative Medicine (ARCCIM), Faculty of Health, University of Technology Sydney. As part of the recruitment for the ACORN PBRN a 21-item practitioner questionnaire was distributed to all registered chiropractors across Australia [18]. While completion of the practitioner questionnaire was a prerequisite for inclusion in the ACORN PBRN database (Of the 2,005 participants reported in this paper 1,680 chiropractors also consented to be included in the ACORN PBRN practitioner database, i.e. 83.8% of responders) [19] it was also possible for chiropractors to complete the practitioner questionnaire but not consent to participate in the ongoing PBRN. The analyses reported in this paper focuses specifically upon the data gathered from the practitioner questionnaires regardless of the responder providing or not providing consent to be included in the ACORN PBRN database.

### Recruitment and sample

Recruitment for the ACORN PBRN including the practitioner questionnaire reported in this paper was conducted between March and June 2015 and consisted of an invitation pack distributed to all registered chiropractors via both professional organisations and a profession-wide mail out based on publically available information (including non-members). The invitation pack was also distributed via a number of regional chiropractic-related conferences and events and was also made available online through the ACORN website during the recruitment period. All participants were offered opportunity to complete the practitioner questionnaire via either online access (SurveyGizmo™) or hard copy. A reminder invitation pack was distributed four weeks following initial invitation pack distribution via the same channels and 4 reminders were sent to potential participants via email where possible. Further details regarding the ACORN PBRN recruitment and promotion strategies can be found elsewhere [19].

At the time of recruitment there were 4,684 registered chiropractors in Australia and 2,005 chiropractors completed the questionnaire providing a response rate of 43%. Of those 1,119 (55.8%) returned a hard copy questionnaire and 886 chiropractors (44.2%) participated in the online survey. In total, 92.8% of respondents were members of one or both of the two major organisations (CAA and COCA) and 2.7% of respondents were not members of either organisation. Compared to the entirety of registered chiropractors as registered by AHPRA in March 2015 [3] the sample of questionnaire respondents has been found to be representative in terms of gender (*p* = 0.634) and age (*p* = 0.065). While the workforce sample is also generally representative of the wider chiropractic population regarding practice location, we found slight differences in terms of the distribution of State of residency with the workforce sample slightly over-represented by chiropractors from South Australia, the Australian Capital Territory, Tasmania and the Northern Territory (*p* < 0.01) [19].

### Questionnaire

The instrument used for this study consisted of a 21-item questionnaire including examination of practitioner characteristics, practice characteristics, and clinical management. The questionnaire was developed by an interdisciplinary ACORN Project Steering Committee and subsequently pilot tested amongst a sample of chiropractors to ensure the final format, content and wording were practitioner-sensitive, readily understood and as timely to complete as reasonably possible.

The chiropractic practitioner characteristics examined included age, gender, number of years in private chiropractic practice, highest level of chiropractic professional qualification attained, professional organisation membership, and roles as a chiropractor in the previous 12 months. The practice characteristics included average patient care hours and patient visits per week, number of practice locations, area of practice location (urban, rural, remote), State/Territory of practice, other health professionals working in their practice location, professional referral relationships (sending and/or receiving referrals), use of diagnostic imaging, and use of electronic records. Clinical management measures included the frequency of discussion with patients regarding lifestyle aspects of care/management plans, frequency with which chiropractors treat people across a range of conditions and broader patient subgroups, and the frequency with which chiropractors employ a range of techniques/methods and musculoskeletal interventions within their patient management.

### Statistical analyses

All data were imported into the statistical software Stata 13.1. Data were checked for plausibility and cleaned for outliers. Data are presented in absolute and relative frequencies for dichotomous or categorical variables, as well as means and standard deviations for continuous variables.

## Results

### Practitioner characteristics

Of the 2,005 chiropractors who participated, 62.4% were male and the average age was 42.1 (SD = 12.1) years. Nearly all chiropractors (97.1%) had a bachelor degree or higher, with the majority of chiropractor’s highest professional qualification being a bachelor or double bachelor degree (34.6%), followed by a Master’s degree (32.7%), Doctor of Chiropractic (28.9%) or PhD (0.9%). Only a small number of chiropractor’s highest professional qualification was a diploma (2.1%) or advanced diploma (0.8%). The average number of years in practice was 15.8 (SD = 11.3) years.

Other than private practice, the respondents report being involved in a number of other roles as a chiropractor in the previous 12 months, including: university teaching (7.2%), research (6.0%), clinical supervision (10.5%), volunteer work (18.3%), and professional organisation activities (19.4%).

### Practice characteristics

The majority of chiropractors are based in (the State of) New South Wales (34.4%), followed by Victoria (24.3%), Queensland (14.5%), Western Australia (13.3%), South Australia (8.9%), Australian Capital Territory (2.3%), Tasmania (1.5%), and the Northern Territory (0.8%). Overall 7.3% of chiropractors routinely consulted patients in a language other than English, with 61.4% of those chiropractors consulting in European languages, and 36.6% in Asian languages (Multiple languages possible). It should also be mentioned that 1.4% of those chiropractors consulted in some sign languages.

Most chiropractors are located in an urban area (73.6%); with 75.1% of chiropractors routinely consulting in one location only. Of those who practice in more than one location, 80.9% practice in two locations and 13.3% practice in 3 locations.

The majority of chiropractors (78.1%) practice in a multi-practitioner location, with 46.0% of all chiropractors working with one other health practitioner, and 19.2% working with two other health practitioners (Table 1) with the most common ‘other practitioner’ type being another chiropractor (56.6%), a massage therapist (29.6%) or a psychologist/counsellor (12.0%). In terms of professional referral relationships, the chiropractors report sending and/or receiving referrals from GPs (55.1%) followed by podiatrists (38.5%), and physiotherapists (30.5%), see Table 1.

**Table 1:** Health care practitioners located in the same practice location and health practitioners in a professional referral relationship with chiropractors

Health Care Practitioner	Working in same practice location	Professional referral relationship (sending and/or receiving)
	%	%
Chiropractor	56.6	-
Massage therapist	29.6	12.9
Naturopath	10.8	6.1
Podiatrist	9.1	38.5
Exercise physiologist	6.3	15.1
Physiotherapist	9.1	30.5
Psychologist/counsellor	12.0	13.8
GP	6.2	55.1
Medical specialist	2.7	15.5
Other(s)	29.2	20.9
None	21.9	23.4

The respondents report spending an average of 27.3 (SD = 12.6) hours per week on patient care and providing an average of 87.3 (SD = 57.7) patient visits per week. Diagnostic imaging is utilised as part of practice by 47.7% of the chiropractors on an ‘often’ basis, and 39.6% of the chiropractors on a ‘sometimes’ basis. One in five (20.2%) of the chiropractors reported having imaging facilities or scanning tools available on site, including X-ray (14.9%), MRI (3.2%), surface electromyography (SEMG) (4.1%), thermography (4.5%), ultrasound (2.8%), and other (4.3%). Electronic records are used by 41.7% of all respondents, with 33.0% of all the chiropractors using electronic records for initial history, 39.4% for subsequent patient visits, and 34.1% for examination findings.

### Clinical management

The chiropractors report discussing a range of topics as part of their care/management plans (Table 2). The most common topic discussed on an ‘often’ basis was physical activity (84.9%), followed by diet/nutrition (50.5%) and occupational health and safety (40.9%). Chiropractors treat people presenting with a variety of conditions with low back pain (axial) (94.7%), neck pain (axial) (93.6%), and headache disorders (87.2%) being the most common conditions treated on an ‘often’ basis (Table 2). In terms of subgroups of patients treated by chiropractors, 73.5% of chiropractors treat older people (≥65 years) on an ‘often’ basis, 53.2% treat children (4–18 years) on an ‘often’ basis, and 49.5% treat athletes or sports people on an ‘often’ basis (Table 2).

**Table 2:** Clinical management characteristics including components of the clinical care/management plan, subgroups of patients and conditions they present with at the chiropractors

Clinical management	Percent often
Discuss	
Physical activity	84.9
Diet/nutrition	50.5
Occupational health and safety	40.9
Nutritional supplements	37.1
Smoking/drugs/alcohol	24.8
Pain counselling	24.6
Medications	23.1
Patient subgroups	
Older people (65 years and over)	73.5
Athletes or sports people	49.5
Children (4 to 18 years)	53.2
Pregnant women	36.7
People with work-related injuries	36.2
Children (up to 3 years)	30.1
People with traffic-related injuries	13.7
Non-English speaking ethnic groups	6.5
People receiving post-surgical rehabilitation	6.4
Aboriginal and Torres Strait Islander people	1.8
Presenting condition(s)	
Low back pain (axial)	94.7
Neck pain (axial)	93.6
Headache disorders	87.2
Thoracic pain (axial)	84.7
Low back pain (referred/radicular)	80.9
Spinal health maintenance/prevention	73.1
Degenerative spine conditions	64.8
Neck pain (referred/radicular)	62.8
Upper limb musculoskeletal disorders	62.6
Postural disorders	61.3
Lower limb musculoskeletal disorders	60.4
Migraine disorders	53.0
Thoracic pain (referred/radicular)	46.4
Non-musculoskeletal disorders	30.0

Numbers indicate percentage of chiropractors who discuss those topics/treat those patients on an often basis

The chiropractors report employing a number of techniques and methods in their patient management (Table 3). The most common techniques/methods employed on an ‘often’ basis are: high velocity, low amplitude adjustment/manipulation/mobilisation (82.2%); extremity manipulation (58.8%); drop-piece techniques/Thomson® (53.7%); and instrument adjusting (52.3%). Chiropractors also use a range of musculoskeletal interventions in their patient management, with the most common intervention used on an ‘often’ basis being soft tissue therapy, trigger point therapy, massage therapy and/or stretching (66.1%) followed by specific exercise therapy/rehabilitation/injury taping (49.3%) and heat/cryotherapy (16.6%) (Table 4).

**Table 3:** Clinical management characteristics including chiropractic techniques and methods, and musculoskeletal interventions used by chiropractors

Clinical management	Percent often
Techniques/Methods	
High velocity, low amplitude adjustment/manipulation/mobilisation	82.2
Extremity manipulation	58.8
Drop-piece techniques/Thomson® or similar	53.7
Instrumental adjusting	52.3
Biomechanical pelvic blocking/sacro-occipital technique®	44.0
Applied Kinesiology® (AK)	16.2
Functional neurology	13.3
Flexion-distraction	8.0
Chiropractic BioPhysics®	4.4
Musculoskeletal intervention	
Soft tissue therapy, trigger point therapy, massage therapy, stretching	66.1
Specific exercise therapy/rehabilitation/injury taping	49.3
Heat/cryotherapy	16.6
Dry needling or acupuncture	13.7
Orthotics (foot care)	10.1
Electro-modalities (eg, TENS)	9.8

Numbers indicate percentage of chiropractors who discuss those topics/treat those patients on an often basis

**Table 4:** Selected chiropractors’ characteristics per practice in different states/territories. Numbers in percentage of chiropractors in each state/territory

	NSW	VIC	QLD	WA	SA	TAS	NT	ACT
	*n* = 680	*n* = 479	*n* = 479	*n* = 263	*n* = 175	*n* = 29	*n* = 15	*n* = 46
Gender								
Male	65.1	59.2	62.5	59.0	65.1	69.0	86.7	52.2
Female	34.9	40.8	37.5	41.0	34.9	31.0	13.3	47.8
Patient care								
Hours, Mean ± SD	27.7 ± 11.5	26.5 ± 10.9	28.9 ± 9.7	24.1 ± 10.6	27.4 ± 9.3	25.5 ± 13.0	30.5 ± 7.7	24.7 ± 8.6
Visits, Mean ± SD	72.9 ± 51.9	86.6 ± 57.3	107.1 ± 58.4	94.1 ± 64.1	105.3 ± 53.5	97.2 ± 70.5	112.9 ± 40.8	73.3 ± 42.0
Area								
Rural	74.1	69.3	68.0	79.7	69.2	48.5	50.0	95.8
Rural	7.9	12.4	13.7	7.5	14.1	24.2	21.4	4.2
Remote	18.0	18.3	18.3	12.8	16.7	27.3	28.6	0.0
Qualification								
Diploma	3.1	1.7	2.5	0.0	4.1	3.6	0.0	0.0
Advanced Diploma	0.7	0.8	0.7	0.8	0.6	0.0	0.0	2.2
Doctor of Chiropractic	13.8	57.6	32.0	56.9	26.2	42.9	26.7	28.3
Bachelor	38.4	17.8	33.5	20.0	32.0	32.1	46.7	32.6
Master’s degree	43.9	22.0	31.3	22.3	37.2	21.4	26.7	37.0
PhD	1.0	0.8	0.7	0.4	0.6	0.0	0.0	0.0
Use of electronic records	45.6	46.4	51.9	43.4	29.1	20.7	60.0	39.1
Use of imaging, often	46.9	45.8	55.1	54.4	42.3	37.9	46.7	23.9
Referrals								
GP	58.7	55.1	57.1	55.1	54.9	72.4	60.0	50.0
Medical Specialist	18.8	18.4	10.1	10.3	10.3	13.8	33.3	19.6
None	20.3	24.0	22.0	20.2	16.6	24.1	26.7	17.4
Consulted in language other than English	10.0	7.9	3.8	3.8	4.6	0.0	6.7	4.3

## Discussion

This is the first in-depth chiropractic workforce survey drawing upon a nationally representative sample of Australian chiropractors. This survey not only confirms basic findings from prior enquiries but also reveals a number of new and significant insights.

Our study findings add further weight to evidence that the national chiropractic workforce is becoming increasingly female over time. This trend was highlighted in a previous study [17], and seems to be further reflected in our more recent data. The chiropractic workforce is well educated with almost all practitioners having undertaken university training. This is not too surprising given that registration as a chiropractor in Australia requires completion of an accredited chiropractic program which is provided at public universities in Victoria, New South Wales, Western Australia and Queensland [20].

It is noticeable however that only 0.9% of chiropractors in our study currently have a PhD. This level of PhD award represents only a small increase compared to the 0.7% identified in 2010 [17] and while comparable to other allied health care professions such as physiotherapy [21, 22] this finding does appear to indicate a somewhat limited capacity to conduct and lead research as well as supervise future chiropractic-focused PhDs within the current Australian chiropractic workforce [16]. The need for strengthening chiropractic research in Australia has been noted previously [23], and several steps have been proposed to generate and encourage chiropractic research capacity in Australia in the face of much needed improvement in research output and activity from within Australian chiropractic university departments [24]. Such a proposal is supported by findings that chiropractors do educate themselves using research literature [25], but often feel inadequately trained to conduct clinical research [26, 27]. Perhaps given the lack of PhDs amongst Australian chiropractors as identified in our study a beneficial approach for research capacity building may be to draw upon academics/methodologists beyond chiropractic to at least help lead or co-supervise PhD research [28] — in areas such public health, health services research and clinical research. Such research collaboration beyond the discipline of chiropractic would not only add much needed capacity but also ensure designs are rigorous and critical. The low number of PhD qualifications is countered by the large percentage of Master’s degrees (>30%) in the cohort, as such there is large potential within the chiropractic community to establish significant and sustainable research capacity.

In the context of research capacity building, it is interesting to note that 6% of the chiropractors in our study did report a personal engagement in research activities and while the specific type of research engagement is not known (it may possibly range from conducting or collaborating on a research activity to possibly having been a targeted participant in a research project) this finding does suggest there may at least be a relatively substantial level of research familiarity amongst chiropractors that the profession may be able to build upon for future research capacity building. Further research is required to explore the research-practitioner interface amongst chiropractors including examination of the perspectives and experiences amongst the ranks of the Australian chiropractic profession towards empirical enquiry and an evidence-based platform for practice.

Chiropractors in Australia also report an average of 27 patient contact hours per week across a wide patient age range. Previous reports have found chiropractors working an average of 33 h per week [1, 16], indicating that a substantive proportion of work may not include actual patient contact. Either way, results indicate significantly lower total working hours of chiropractors on average than those reported by other health professions, such as GPs who work around 45 h per week on average [29]. The low number of patient contact hours raises questions as to why chiropractors might be working so little, and more research is needed to determine if it is the chiropractors’ choice, or if it is related to lack of patients, the level of intra- and interprofessional competition, or other unknown reasons which may have significant implications for the future workforce development. Chiropractors in our sample also report consulting an average of 87 patients per week, which equates to a chiropractor spending an average of around 20 min with each patient. An average consultation time that is more than double the reported average consultation time for a GP in Australia [30] but much less than the average consultation time (up to 60 min) identified for other allied health and CAM practitioners [31, 32]. Based on the average patient visit data identified from our study we estimate that Australian chiropractors currently manage around 21.3 million patient visits per year and this figure would suggest chiropractors play a relatively substantial role in healthcare provision in contemporary Australia.

Another interesting finding from our analyses relates to the inter-professional relationship between chiropractors and other health care providers in Australia. While the vast majority of chiropractors in our study report working with another chiropractor or massage therapist in the same practice location, and only a minority of chiropractors report a GP or physiotherapist as working in their practice location, a large number of chiropractors are interconnected to GPs, physiotherapists and other health care professionals by sending and/or receiving referrals. Indeed, a substantial percentage of chiropractors in our study report having a referral relationship with GPs, physiotherapists and other health care professionals. This aligns with previous research that has found more than half of GPs regularly refer to chiropractors [33, 34] and that chiropractors understand and value the importance of referral relationships with other professions [35]. Some aspects of such referrals may also be related to health insurance, as chiropractic care is eligible for allied health services coverage (via Medicare, the Australian publicly funded universal health care scheme) if it is directed by a medical referral to assist chronic disease management [14]. However, recent findings indicate that only a minority of patients utilise chiropractic care via Medicare coverage [7]. Unfortunately, our study did not identify the direction of referrals between the chiropractors and GPs, and further research is warranted to explore both who initiatives referrals between GPs and chiropractors and the relative experiences of the two parties regarding these patient referrals with a view to helping facilitate more effective inter-professional collaboration. Further research is also required to examine the interplay between referral and the use of electronic records. The low number of chiropractors using electronic health records may negatively impact potential referral relationships, as well as future educational and training needs.

The vast majority of chiropractors report being consulted by patients with musculoskeletal conditions such as back and neck pain, which is in line with the traditional chiropractic focus on spinal health [36, 37]. It can also be assumed that the high percentage of thoracic spine conditions that patients present with is related to lifestyle factors and desktop workplace setups [38], and requires significant efforts in examining efficacy and safety of chiropractic treatments for those conditions. Accordingly, chiropractors use a broad range of manual techniques and musculoskeletal interventions suited for the treatment of back and neck pain. Of note is the finding from our study that chiropractic patient management includes a wide range of health advice on diet and nutrition, physical activity, minimising alcohol and smoking and drug consumption. These findings highlight the role and focus of chiropractors toward disease prevention and health promotion as reported in other studies [39–42] and there is a need to further assess the whether the chiropractic workforce may represent a valuable resource for promoting wider health promotion and prevention in Australia [43, 44].

Our findings may be limited due to several factors. The survey data was collected via self-report and this may have introduced a possible recall bias. Furthermore, the depth to which our questionnaire examined some workforce issues was further limited in view of encouraging a reasonable response rate and some of these issues will require follow-up in future research. Due to space limitations for example no detailed information on education and trainings, ethnicity, and attitudes and beliefs of chiropractors were explored; those issues will be examined in further sub studies. Nevertheless, despite these limitations, previous workforce surveys of Australian chiropractic have reported substantially lower response rates [17], and no previous research has attempted to attract a national representative sample of chiropractic care [17, 40]. The workforce survey reported here therefore comprises one of the largest scale voluntary workforce samples in the field of chiropractic or allied health care to date.

## Conclusion

The chiropractic profession represents a substantial component of the contemporary Australian health care system. While the Australian chiropractic workforce is well educated, research engagement and capacity remains poor and there is much room for future initiatives to help chiropractic reach full potential as a key integrated profession within an evidence-based health care system. Further rich, in-depth research is required on a number of specific topics relating to the practice of chiropractors in Australia in order to ensure safe, effective and coordinated health care for all patients.
